# Evolution of the Study of Phase Diagram of Binary and Ternary Mixtures Involving Fatty Acid Esters

**DOI:** 10.3390/ma14020369

**Published:** 2021-01-13

**Authors:** Gabriel Rubio-Pérez, Natalia Muñoz-Rujas, Fernando Aguilar, Rebecca Ravotti, Lukas Müller, Eduardo Montero

**Affiliations:** 1 Escuela Politécnica Superior, Universidad de Burgos, Avenida Cantabria s/n, 09006 Burgos, Spain; grubio@ubu.es (G.R.-P.); faguilar@ubu.es (F.A.); emontero@ubu.es (E.M.); 2 Competence Centre Thermal Energy Storage (TES), Lucerne University of Applied Sciences and Arts, 6048 Horw, Switzerland; rebecca.ravotti@hslu.ch (R.R.); lukas.mueller@hslu.ch (L.M.); 3 EaStCHEM, School of Chemistry, The University of Edinburgh, Edinburgh EH9 3FJ, UK

**Keywords:** thermal energy storage, phase change materials, phase diagrams, fatty acid esters, review

## Abstract

Interest in phase change materials keeps on rising as thermal energy storage grows in popularity in the scientific community as a promising complement for renewable energies in the future. Extending the possibilities beyond pure compounds, the use of mixtures (especially eutectics) widens the range of suitable phase change materials (PCM) available in the market. However, a precise knowledge of the mixtures’ phase behavior is required, making phase diagrams the most appropriate tools to follow. The aim of this work is to collect and analyze published literature concerning the phase diagrams of fatty acid esters mixtures, which constitute promising candidates as PCM due to their attractive properties, such as high latent heat, chemical stability and the possibility of extracting them from vegetable and animal oils. The topic appears as a still open scientific field, where further studies need to be performed to complete, complement and perfect the currently available information.

## 1. Introduction

Horizon 2020 is coming to an end, while Horizon Europe is already taking over. In the new framework proposed, the topics of energy, natural resources and environment are present in two of the six clusters included in the second pillar of the program, and therefore are still strongly considered in the guidelines suggested [1]. In the same line, as 2020 passes by, the fifth anniversary since the signing of the Paris Agreements is celebrated, thanks to the efforts of organizations such as the International Energy Agency (IEA), who has taken seriously the climate emergency and led the challenge of setting the bases for a greener universal energy system. Thus, it can be seen that energy efficiency remains as a priority in the current global trend towards more environmentally-friendly energy generation. Recent scientific advances have allowed a continuous improvement of renewable energies, but other techniques towards a more efficient energy use have also experienced a remarkable boost, such as electric batteries and zero energy buildings.

Thus, energy storage is gaining importance in the scientific panorama as a complementary technique to renewable energies capable of bridging the gap of availability and demand. In this line, thermal energy storage (TES) is gradually becoming an important supplementary technique to, for example, solar energy, where a considerable amount of energy is generated in the form of heat but usually wasted. In a residential building, this energy can be stored and used as a source of heat for space heating and domestic hot water (DHW) during the night, when the photovoltaic panel is not able to generate energy [2].

Latent heat thermal energy storage (LTES) represents one of the ways (along with sensible and thermochemical energy storage) through which heat can be stored for its later usage in thermal energy storage setups. This is why phase change materials (PCM), compounds with a key role in LTES, become important to achieve more efficient energetic system. A recent overview on the state-of-the-art of TES, especially LTES and PCM, is found in the works of Jouhara et al. [3] and Elias et al. [4]. Important innovative milestones are currently being reached towards a better management of concentrated solar power plants through the use of PCM [5,6,7]. Other fields are also incorporating the use of these materials and their mixtures with other compounds allowing, for example, the generation of new composite materials [8,9,10] or improving the thermal performance of buildings [11,12,13]. Thus, the relevant advantages of PCM such as thermal inertia and energy storage capabilities are incorporated in traditional products, generating innovative and improved solutions.

Several studies have been published concerning what kind of materials can be used efficiently as PCM depending on the application [14,15,16]. Understanding deeply the specific properties and behavior of each of these materials is fundamental to properly choose the most suitable one depending on the purpose and conditions of the final application. There is a wide range of compounds used as PCM, from paraffins to salt hydrates. Some of these compounds, fatty acid esters (FAE), present interesting advantages, such as high phase change enthalpy, low supercooling, chemical stability, low corrosivity and the possibility of forming eutectics or being obtained by natural means [17,18,19]. This is why these compounds are gaining interest as potential PCM in the scientific community.

When designing a LTES system, the proper understanding of the thermal substance’s behavior is an essential part of the process, which becomes especially relevant when mixtures instead of pure substances are being used as PCM. The main reason for using mixtures as PCM is to take advantage of the formation of eutectics, that is, specific molar fractions of mixtures that behave as pure substances but with lower melting temperatures [20]. This considerably expands the possibilities when choosing a PCM for a certain application, thus widening the range of phase change temperatures available. The properties of pure compounds are extensively reported in the literature and reference books [21,22,23], but, when mixing two, three or even more pure elements to form a mixture, the behavior becomes more complex. This is due to the fact that the thermophysical properties of mixtures are strongly dependent on the molar fraction of the primitive compounds, therefore a more precise knowledge is required to characterize properly the materials. In these cases, a useful tool is the phase diagram, in which the phase change temperature is plotted versus the molar fraction, showing all the different phase transitions that occur during melting through the whole range of concentrations of the mixture [20].

Abundant literature is found concerning the study of phase diagrams of, for example, fatty acids [24,25,26], but this information is less common for FAE. Mixtures of common fatty acid ethyl and methyl esters have been reported, but in most cases only the liquidus line (temperature in which solidifications starts) is shown, while no information is given about the different phases present in the solid region and other transitions such as peritectics and metatectics [27,28,29,30,31,32,33,34]. This lack of publications is more evident when it comes to mixtures of these compounds with other kind of substances, such as alkanes, aromatics and fatty acids. These types of blends are starting to gain interest as potential PCM suitable for LTES, but the absence of reported research represents an important barrier yet to be overcome.

To the best of our knowledge, no previous review articles concerning specifically the study of phase diagrams of binary and ternary mixtures of FAE have been found, although interesting summaries of the reported literature on this are found in articles from Robustillo et al. [35] and Branco et al. [36].

Based on this, the aim of this paper is to provide a brief but comprehensive review on all the literature published thus far concerning the study of phase diagrams of mixtures involving fatty acid esters. The intention of this work is to provide useful references to researchers willing to study the mixing behavior of FAE as potential phase change materials and to offer a critical analysis on the trend of the information reported thus far in this scientific field. This information will be relevant to researchers on TES but also experts in other fields, such as the thermal management of batteries or the development of new construction materials.

This paper is structured as follows. Section 2 describes in an ordered manner and in detail all the literature available on the topic, divided in subsections according to the kind of substances present in the mixture. In Section 3, some reflections are suggested and discussed based on a rational criterion to provide an outlook to the overall situation. Finally, in Section 4, some conclusions are extracted based on all the analysis performed.

## 2. Review on Published Literature Concerning Phase Diagrams of Mixtures of Fatty Acid Esters

In this section, articles previously reported on the study of phase diagrams of binary and ternary mixtures of compounds involving fatty acid esters are presented, described and analyzed, divided into the four following subsections: mixtures of fatty acid ethyl esters (FAEE), mixtures of fatty acid methyl esters (FAME), mixtures of FAEE with FAME and mixtures of fatty acid esters with other compounds.

Table 1 summarizes the main information found in each of the described references following a chronological order, namely measured mixtures, defined phase transitions (melting, eutectic, peritectic, etc.), reported information (solid–liquid equilibrium data, phase diagrams, Tammann plots, etc.) and theoretical and predictive models employed. The meaning of each one of the abbreviations used in this table is reported in the section “Abbreviations” at the end of the article. Table 2 classifies the references based on the research’s main purpose, thus showing the authors’ motivation behind each one of the presented works.

Table 3 intends to support those researchers seeking suitable mixtures of FAE for specific applications. In this table, the references are ordered according to the melting temperature at the eutectic concentration, in ascending order from the lowest to the highest temperature, as the eutectic is usually the interesting concentration of the mixture to work with as PCM. Some considerations have been taken into account when creating this table:

- Mixtures in which there is no formation of eutectic points due to the chemical behavior of the materials in the mixture are not reported.
- When eutectic temperatures are not specified by the authors, the lowest melting temperature is considered as the temperature of the eutectic because of the very definition of the eutectic point.
- When temperatures are reported originally in Celsius, they are here reported in Kelvin (considering that 0 °C = 273.15 K) to unify the style of the table and facilitate its use.
- Numbers extracted approximately from a graph because no numerical data are reported by the authors are marked with an indicator (*).
- Ternary mixtures are not reported.
- When the melting temperatures of the mixture’s materials is so high that the eutectic data correspond to the data of one of the pure materials, it is indicated in the “Comments” column of the table.
- The values of enthalpy are reported, when available, in the same units provided by the authors.

### 2.1. Mixtures of Fatty Acid Ethyl Esters

Asides from an article from Lutton et al. published in 1962 [38], more recently mentions of binary mixtures involving two FAEE are found in the work of Suppes et al. [29], in which several mixtures of FAME and FAEE are studied in six different and equidistant molar fractions, with the mixture ethyl palmitate and ethyl stearate being one of them. Experimental points are compared to the theoretical model of the liquidus line, with the purpose of evaluating the potential of these mixtures as phase change materials.

Despite focusing more on a better understanding of biodiesel rather than on thermal energy storage, a first thorough review study is that of Lopes et al. [32], mainly on the binary mixtures of ethyl laurate with ethyl myristate, ethyl palmitate and ethyl stearate. Despite the lack of data at that time concerning FAEE, the literature is collected and correlations are proposed for the theoretical determination of the phase change temperature and enthalpy of the compounds. Using the UNIQUAC model to describe the activity coefficients of the solid phase, a predictive model is proposed to estimate the cloud point of the mixtures based on the work previously developed by other authors for alkanes [60] and fatty acids [61]. This is then compared to the experimental results, which are measured using a DSC.

The same model was used by Boros et al. [34], whose research focused on FAEE with the idea of studying the behavior of biodiesel as well. The binary mixtures of ethyl stearate with other seven FAEE are studied in terms of the cloud point, comparing predictive models with experimental results. A similar approach was followed by Costa et al. [42], in which the cloud point of six binary mixtures of ethyl palmitate with different FAEE is studied.

**Figure 1:**
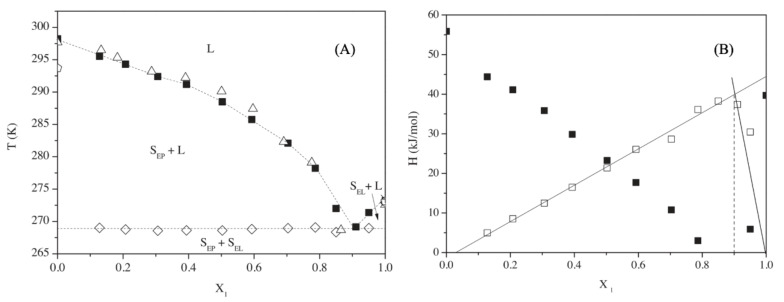
Examples of (**A**) a phase diagram and (**B**) a Tammann plot of the mixture ethyl laurate/ethyl palmitate [45]. In Figure 1A: ◼ ⎯ liquidus line from [45]; △ ⎯ liquidus line from [42]; ◇ ⎯ Eutectic temperature; L ⎯ Liquid; SEP ⎯ Solid Ethyl Palmitate; SEL ⎯ Solid Ethyl Laurate. In Figure 1B: ◼⎯ Melting; ☐ ⎯ Eutectic. Reprinted from Fluid Phase Equilibria, 358, Maria Dolores Robustillo, Deise Fernanda Barbosa, Antonio José de Almeida Meirelles, Pedro de Alcântara Pessôa Filho, “Solid–liquid equilibrium in ternary mixtures of ethyl laurate, ethyl palmitate and ethyl stearate”, 272–281, Copyright (2013), with permission from Elsevier.

Several relevant studies on the solid–liquid equilibrium of ternary mixtures of FAEE were carried out for the first time by researchers from the University of São Paulo, and the results of the binary mixtures were reported too. Important novelties are adopted in these works. Firstly, the peak temperature of the DSC curve is considered as the most representative point to define the phase transition, a detail unspecified in the previously described works. In addition, unlike in previous studies, more complex phase diagrams than the simple eutectic supposed thus far are considered likely to occur. That is the reason further investigations with other measuring devices (apart from the DSC) are recommended. In [35], the experimental points for the liquidus line are reported, but eutectic and other measured transitions are presented too. Up to four different solid phase transitions were measured by Robustillo et al. [45] in the mixture ethyl palmitate with ethyl stearate. Tammann plots, in which the phase change enthalpy versus molar fraction is represented, are also found for the first time for these kinds of mixtures. Examples of both a phase diagram and a Tammann plot of one of the reported mixtures, ethyl laurate/ethyl palmitate, are shown in Figure 1.

This work progression reaches its summit in [46,47], where several transitions are reported (eutectic, peritectic, metatectics and others) and a potential interpretation of the different solid phases below the liquidus line is proposed based on the experimental results and a thorough analysis of the DSC curves.

All the previous research performed by the aforementioned authors is collated in [49] and completed with the depiction of nine new solid–liquid phase diagrams involving ethyl caprylate, ethyl caprate, ethyl oleate and ethyl laurate. The experimental results from both this study and the previous ones, which are compared with the liquidus line of the ideal solution predicted by the previously proposed UNIQUAC method, show a good adjustment. The Tammann plots are also reported.

All the mixtures mentioned were measured at atmospheric pressure. Only Carareto et al. [37] reported the cloud point of three binary mixtures of FAEE at higher pressures. Here, an experimental setup with a high-pressure cell is utilized to measure the melting points of the mixtures at 20, 40, 60 and 80 MPa, while a regular DSC is employed to measure them at atmospheric pressure. Thus, the effect that the increase of the pressure has on the temperature displacement of the cloud point is evaluated.

All the references mentioned in this section are gathered in Table 4 to allow the reader to find with ease the articles in which a specific mixture of two fatty acid ethyl esters is described. This table contains only the binary mixtures. The only four ternary mixtures reported are: ethyl oleate/ethyl laurate/ethyl palmitate [35], ethyl laurate/ethyl palmitate/ethyl stearate [45], ethyl laurate/ethyl palmitate/ethyl myristate [46] and ethyl oleate/ethyl myristate/ethyl stearate [47].

### 2.2. Mixtures of Fatty Acid Methyl Esters

A similar tendency to that of fatty acid ethyl esters’ mixtures is observed concerning the study of phase diagrams of binary mixtures of FAME through the years. Some references from the second half of 20th century can be found [38,39,40], and some binary mixtures of FAME were reported in one of the articles already mentioned [29], but that of Imahara et al. [31] can be considered the first work specifically studying the cloud point of binary mixtures of fatty acid methyl esters. The solid–liquid equilibrium of eight mixtures is reported, and the cloud point line is predicted using the same model found in [29]. Several mixtures of the compounds studied are prepared at different molar fractions to simulate the behavior of an actual biodiesel, and the cloud point results obtained are compared to those of some common biodiesel fuels extracted from different feedstocks such as sunflower, soybean and palm.

The work of Lopes et al. [32] has also already been mentioned, but here the cloud point of a binary mixture of fatty acid methyl esters is also mentioned, following the same structure as for the study of fatty acid ethyl esters. The experimental cloud point results are reported alongside those from the predictive model based on UNIQUAC.

Three binary mixtures of FAME were measured and reported by Costa et al. [41]. In this work, phase diagrams are proposed for the studied mixtures, including all the transitions extracted from the DSC curves (melting, eutectic, peritectic, metatectic and other transitions). Unlike previous studies, here an optical microscope with polarized light is also used to observe the phase change process and its morphology. This allows the design of more complex phase diagrams, as both physical and optical information is used to specify what phase change is occurring and when. Tammann plots are also reported.

Only one binary mixture was studied by Xu et al. [48], who aimed not to evaluate the behavior of the fatty acid methyl esters in a biodiesel as previous authors did, but rather to study the potential of the binary mixture methyl palmitate/methyl stearate as a base PCM to develop a novel composite phase change material (CPCM).

The same purpose leads the research by Liston et al. [50], who studied two binary mixtures with eutectic temperatures between 0 and 3 °C considering their possible incorporation into concrete pavement to improve its behavior in winter conditions and reduce the accumulation of snow and ice. Using only a DSC, the onset temperature is considered the melting temperature, while the peak temperatures are used for the other transitions happening during the phase change. Experimental points, phase diagrams and Tammann plots are reported for both mixtures.

All the references mentioned in this section are collected in Table 5 to allow the reader to find with ease the articles in which a specific mixture of two fatty acid methyl esters is described.

### 2.3. Mixtures of Fatty Acid Ethyl Esters with Fatty Acid Methyl Esters

Binary mixtures involving both a FAEE and a FAME have not been studied as extensively as the mixtures previously reported, and the literature is scarce.

Some examples of such mixtures were reported by Lutton and Hugenberg [38] and Suppes et al. [29], but Maximo et al. [56] can be considered the only authors specifically researching mixtures of fatty acid ethyl esters with methyl esters. In the line of previous works from the same authors [34,41,42], three binary mixtures are reported, including the experimental results, phase diagrams, Tammann plots and optical information of the mixtures during the phase change. The main purpose of this paper is to study the potential use of blends of these substances to reduce the cloud point of biodiesels.

All the references mentioned in this section are compiled in Table 6 to allow the reader to find with ease the corresponding articles in which a specific mixture of a FAEE with a FAME is described.

### 2.4. Mixtures of Fatty Acid Esters with Other Compounds

Mixtures of FAE with other kinds of substances were first used by Lobbia et al. [27,28] to obtain the interchange parameters of octacosane and hexadecane by means of group interaction statistics. The experimental solid–liquid equilibrium temperatures are reported without any kind of phase diagram, as this is not the purpose of the study. In [30], the solid–liquid equilibrium of two binary mixtures involving benzene and p-xylene with EM are studied and compared with the models proposed in the article, the main point of the work.

Concerning mixtures of FAME with heavy alkanes and aromatic compounds, important results were reported by the Hungarian research group of Benziane et al. [43,44], with drafting the phase diagram being their main goal. The mixtures are studied as simple eutectic systems, although some other solid–solid transitions are reported too, and the results are compared with those from different predictive and theoretical models.

Relevant results in this field were also reported by Robustillo’s Brazilian team. In [52], the ternary mixture ethyl laurate/ethyl palmitate/dodecylcyclohexane is studied. Two different methodologies are proposed for the determination of its polymorphism, and the experimental results of each of the three binary mixtures of these compound are reported too. In addition, different models are considered to evaluate and predict the behavior of the liquid phase. A similar structure was followed by Robustillo et al. [53] to study the mixture ethyl laurate/ethyl palmitate/decane and Robustillo et al. [57], who mixed five different fatty acid ethyl esters with hexadecane. Single eutectic systems are initially foreseen, but the experimental results show that the phase diagram of these mixtures are more complex than expected.

A ternary mixture involving hexadecane, together with the corresponding binary ones, were also studied by Moura-Nickel et al. [51], who reported the cloud point of the binary and the phase diagram of the ternary mixtures. The idea of considering the peak temperature as the indicator of the phase transition was followed by these authors and supported by previous works from renowned authors.

In [54], a mixture of methyl palmitate with lauric acid is evaluated as a potential PCM, and the results are complemented with the measurement of other thermophysical properties such as specific heat, thermal conductivity, diffusivity and stability. It is worth mentioning that, to our knowledge, this is the only reported work about a mixture of a FAE with a fatty acid.

The same structure found in the aforementioned work by Benziane et al. [43,44] was followed vy Chabane et al. [55], who reported the phase diagrams and Tammann plots of new binary mixtures involving two FAME and three alkanes (tetradecane, hexadecane and octadecane). The experimental results are also compared with the theoretical ones from different models.

Mixtures involving dodecane and p-xylene with fatty acid ethyl esters were studied and published by Bessa et al. [58,59]. Following the same structure, the solid phase is studied in depth, with eutectic and other transitions being reported, in contrast with the previous work of Collinet and Gmehling [30], who also studied p-xylene. The deconvolution of polymorphic curves is also considered in these works.

Finally, an extensive study was recently presented by Branco et al. [36], who studied six binary mixtures of FAEE with alkanes by means of a DSC. Further investigations are carried out through the use of optical microscopy and X-ray diffraction as well, which allows more complex and precise phase diagrams do be developed.

All the references explained in this section are compiled in Table 7 to allow the reader to find with ease the articles in which a specific mixture of a fatty acid esters with another kind of compound is described. This table contains only the binary mixtures. The only three ternary mixtures reported are: ethyl palmitate/ethyl stearate/hexadecane [51], ethyl laurate/dodecylcyclohexane/ethyl palmitate [52] and decane/ethyl laurate/ethyl palmitate [53].

## 3. Discussion

Based on the tendencies of the scientific community when researching and studying binary mixtures of fatty acid esters, especially considering the depiction of phase diagrams, some observations can be made from a general point of view.

The first studies on the topic appear in the 1960s [27,28,38]. Using more traditional methodologies to measure the melting temperature, the results are presented in a schematic way, and only a phase change temperature is reported, namely the melting temperature. This is especially true when the aim of the studies is not the in-depth knowledge of phase change behavior. Over time, less common mixtures are studied to try to bridge the gap in the published data between, and the phase diagrams grow in complexity. This is possible thanks to the extended use of the differential scanning calorimetry technique, the prevalent measuring device in the majority of the articles. At first, the mixtures of these compounds are considered to behave as single eutectic systems, with only the phase transition corresponding to the cloud point [29,31,32,34,42,43,44]. However, soon researchers started to realize that it is possible that the single eutectic system’s assumption could not be representative of the real behavior of these mixtures [32,35,42,45,46,47,52,53,57]. Looking at the DSC curves, more than two peaks are observed, which could be an indication of the sample undergoing multiple phase transitions. The authors realized that the use of DSC could not be enough by itself to characterize these mixtures, as some relevant phase change processes may be not properly visible in the DSC curves. Thus, other complementary techniques started being utilized, such as optical microcopy [41,56], X-ray diffraction (XRD) [36] and Fourier-Transform Infrared spectroscopy (FTIR) [48], allowing more precise and realistic phase diagrams to be developed. The improvement of the measuring devices also allows the management and reporting of additional data, which enables, for example, the representation of Tammann plots [26,32,34,35,42,43,47,49,50,52,54,55,56].

Having a better understanding of the behavior of biodiesel is the main goal of most of the literature reported, as a proper knowledge of the basic compounds’ cloud point leads to a well-known characterization of the global system [32,34,36,41,49]. Nowadays, however, in correlation to the terms “phase diagram”, “eutectic” and “fatty acid esters”, the topic of thermal energy storage is increasingly relevant to researchers in the scientific community as these compounds are becoming more and more popular as potential PCM. Nevertheless, as can be seen, only a few papers, most of them from the last decade, are centered on the use of the studied fatty acid esters as phase change materials [29,48,50,54]. Thus, two different tendencies on the main goal of the research can be observed: on the one hand, the investigation carried out on the study of biodiesels by remarkable researchers from the same team (e.g., Costa, Meirelles, Coutinho and Robustillo), and, on the other hand, a small number of researchers from all over the world concerned about the potential use of FAE in thermal energy storage. This dual path explains the reporting of only the cloud point, which is relevant to biofuels, in most of the older papers, and the measurement of other properties in recent articles, such as the thermal conductivity or cycling stability (useful PCM properties when designing a TES system) [48,54].

In this effort to properly characterize promising PCM involving FAE, suggestions on how to implement the use of these materials as PCM are offered by the few authors researching on this topic. In [29], several mixtures are studied with the general aim of evaluating their potential use as PCM for TES systems. As this paper was published in 2002, this topic was still a novelty, especially when studying less common materials used as PCM, such as FAE. A more specific purpose motivated Xu et al. [48], who developed a novel composite PCM to be integrated in building materials in warm areas of China. Thermal regulation of buildings was also the aim of Saeed et al. [54], but Liston et al. [50] pursued a more innovative application of PCM, that is their integration in pavement concrete to speed up ice melting during winter months.

As can be seen, FAE are attractive materials for thermal energy storage applications, but further research can still be done. As other kinds of materials are becoming popular as PCM in a wide variety of applications [62], future lines of investigation are needed to evaluate the potential use of FAE as PCM. This scientific field is still open to researchers willing to propose and evaluate new ideas.

Concerning the information reported in the literature, a trend in the measuring techniques can be observed. Due to the importance of fatty acid esters in the field of fuels, the cloud point was the important temperature to measure at first, as it indicates the temperature below which wax is formed in diesel, which may provoke clogged filters or the formation of emulsions. That is the reason that, when extracting results from the DSC thermograms, the onset temperature is considered the most suitable one to be taken as the start of the phase change [43,44]. However, as DSC curves are studied more in depth, the phenomenon of overlapping peaks requires a new criterion for the data analysis, and the peak temperature is considered appropriate to indicate transitions such as eutectics and peritectics [35,45,46,47,49,50]. This temperature shifts when the heating rate is changed, but the onset temperature loses significance when calculated in overlapping peaks. Different methods are being developed to generate more precise results, such as peak deconvolution [52,53]. Nevertheless, there is still nowadays a disagreement on which is the most relevant temperature for each phase transition when defining the phase diagram, with even some authors reporting the whole range of temperatures in which the phase change occurs [48].

Predictive models are studied and evaluated for the measured mixtures by almost all the aforementioned authors, with the exception of older articles [27,28,29,38,39,40] and in the work of a few more recent authors evaluating the use of FAE as PCM [29,48,50]. Scientists researching on biodiesel are the ones who extensively report the comparison of experimental results with theoretical models, using mainly UNIQUAC [31,32,34,37,42,43,44,49,55], UNIFAC [30,31,52,53,55,58,59], Flory–Huggins (especially Robustillo’s team) [35,45,46,47,52,53,55,58,59] and ideal models, based on Schraeder’s equation [36,43,44,49,52,53,54,55,58,59]. The use of these models allows the description of the mixtures’ phase change behavior using only data from the pure materials, based on thermodynamic equations [35,45,46,47,51,52,53]. However, due to the lack of experimental data concerning the interaction between the molecules of the compounds forming these mixtures, on some occasions, the key parameters of these models were estimated. Despite the variety of models employed, the authors agree that, for these mixtures, the non-ideality of the materials should be considered to minimize the difference between theoretical and experimental results [57,59]. Although important information is reported on the issue, this represents another potential line of investigation that could be followed in the future: a methodological and rigorous evaluation of all the models available to determine the most suitable one for mixtures of FAE, as non-standard criteria on the most appropriate models for these materials is found in the literature.

Most of the binary mixtures involving fatty acid ethyl and methyl esters have already been studied, with the most common compounds of this category employed. However, some techniques such as XRD and FTIR are allowing a more precise definition of the different phases that occur in the mixtures below the liquidus line. This is the reason the field is still open to new research as, in some cases, the explanations given and the assumptions made on the definition of the different phases depicted in the reported phase diagrams are not fully ascertained.

In a similar way, the study of mixtures of fatty acid esters with other kinds of substances is a promising field of investigation. Mixtures of alkanes with FAE have already been reported to contribute to the generation of more realistic models of biodiesel [36,43,44,57], but, concerning phase change materials, other compounds could be blended with FAE to reach useful melting temperatures and the enhancement of some of their properties. As an example, only one reference has been found about mixtures of FAE with fatty acids [54] despite both classes of materials being widely used as PCM. Thus, the issue is still open to new solutions.

It is also worth mentioning that only a few ternary mixtures are reported [35,45,46,47,51,52,53], as the procedure to perform such measurements is more laborious and the definition and depiction of the phase diagram are more complex. However, these mixtures could have interesting enhanced properties due to the characteristics of the compounds that conform it, but almost no information is available in the literature. Mixing fatty acid esters in triads with other type of substances could generate new mixtures with relevant properties for both the biodiesel and the thermal energy storage scientific communities.

## 4. Conclusions

This review on published literature concerning the study of phase diagrams of mixtures involving fatty acid esters shows that a lot of information has been reported on the topic since the 1960s, but, as the interest in the development of new phase change materials keeps on growing, further information on potential suitable substances for this purpose is needed. The understanding and characterization of biodiesel has been the fuel to spark new research in the field of phase behavior of fatty acid esters for some decades. However, although this motivation is still important, the promotion of renewable energies along with the fact that thermal energy storage is gaining presence in the scientific world are giving new reasons to deepen in the topic. The use of eutectics, and not only of pure compounds, widens the range of materials available when it comes to selecting a suitable PCM for a specific application but also requires more precise phase diagrams to know properly how the thermal storage substance will behave.

The improvement of the measuring technologies as well as the development of new methodologies and materials have boosted the publications of fundamental information throughout the years. However, as energetic systems become more complex and delicate environmental decisions have to be made, more and more information is required to reach the objectives defined even by international organizations. Thus, the study of phase diagrams of mixtures of fatty acid esters is still a promising field in which new research should be done, especially concerning mixtures with other compounds, the use of complementary techniques, the evaluation of currently used theoretical models and the measurement of ternary mixtures. All this information will have a key importance in the further development and evaluation of FAE as PCM.

## Figures and Tables

**Table 1:** Summary of the main information reported in all the references described in this paper. The meaning of the abbreviations used can be found in the section “Abbreviations” at the end of the article. All measurements are performed at atmospheric pressure, except in [37]. No common criteria are followed by all authors concerning which temperature defines the phase transition, so no generalization is done.

References	Mixtures	Tran. Reported	Info. Reported	Models
Lutton and Hugenberg [38] (1962)	EP/ES MP/MS ES/MS	Melting	Cloud point data	n/a
Lobbia et al. [27] (1982)	MS/Octacosane ES/Octacosane	Melting	Cloud point data	n/a
Lobbia et al. [28] (1983)	Hexadecane/MP Hexadecane/MN Hexadecane/MS Hexadecane/ES	Melting	Cloud point data	n/a
Dörfler and Pietschmann [39] (1990)	MP/MH MP/MS MP/ME	Melting	DSC curves Cloud point diagram	n/a
Lockemann and Schlünder [40] (1993)	MM/MP	Melting	SLE data Phase diagram	n/a
Suppes et al. [29] (2003)	MP/MS MP/ES MP/EP MS/ES EP/MS EP/ES MO/MP MO/MS	Cloud point	DSC curves Cloud point data	n/a
Collinet and Gmehling [30] (2005)	EM/Benzene EM/p-Xylene	Melting	Cloud point data Cloud point diagram	UNIFAC (Dortmund)
Imahara et al. [31] (2006)	MP/MO MS/MO MP/MLi MS/MLi MO/MLi MP/MS MM/MP ML/MP	Cloud point	Cloud point diagram	UNIFAC Wilson NRTL UNIQUAC
Lopes et al. [32] (2008)	MM/MP EL/EM EL/EP EL/ES	Cloud point	Cloud point diagram	UNIQUAC
Boros et al. [34] (2009)	ECy/ES ECa/ES EL/ES EM/ES EP/ES EO/ES ELi/ES	Cloud point	Cloud point data Cloud point diagram	UNIQUAC
Costa et al. [41] (2011)	MM/MP MM/MS MP/MS	Melting Eutectic Peritectic Metatectic Other transitions	DSC curves SLE data Phase diagram Tammann plots Optical images	n/a
Costa et al. [42] (2012)	EP/ECy EP/ECa EP/EL EP/EM EP/EO EP/ELi	Cloud point	Cloud point data Cloud point diagram	UNIQUAC
Benziane et al. [43] (2013)	Eicosane/MP Tetracosane/MS Octacosane/MS	Melting Eutectic	DSC curves SLE data Phase diagram Tammann plots	UNIFAC (Larsen & Gmehling) UNIQUAC Ideal models
Benziane et al. [44] (2013)	MS/Byphenyl MS/Naphthalene MP/Byphenyl MP/Naphthalene	Melting Eutectic Other transitions	DSC curves SLE data Phase diagram	NRTL Wilson UNIQUAC Ideal models
Robustillo et al. [35] (2013)	EO/EL EO/EP EL/EP EO/EL/EP	Melting Eutectic Other transitions	DSC curves SLE data Phase diagram	Flory-Huggins
Robustillo et al. [45] (2013)	EL/EP EL/ES EP/ES EL/EP/ES	Melting Eutectic Other transitions	DSC curves SLE data Phase diagram Tammann plots	Flory-Huggins
Carareto et al. [37] (2014)	EL/EM EL/EP EM/EP	Melting	Cloud point data Cloud point diagram	UNIQUAC
Robustillo et al. [46] (2014)	EL/EM EM/EP EL/EP EL/EP/EM	Melting Eutectic Peritectic Metatectic Other transitions	DSC curves SLE data Phase diagram Tammann plots	Flory-Huggins
Robustillo et al. [47] (2014)	EO/EM EO/ES EM/ES EO/EM/ES	Melting Eutectic Peritectic Metatectic Other transitions	DSC curves SLE data Phase diagram	Flory-Huggins
Xu et al. [48] (2014)	MP/MS	Melting	DSC curves SLE data Cycling stability FTIR spectra	n/a
Boros et al. [49] (2016)	ECa/ECy EL/ECy EM/ECy EL/ECa EM/ECa ECa/EO ECa/ELi EL/ELi EM/ELi	Melting Eutectic Other transitions	Cloud point diagram SLE data Tammann plots	UNIQUAC Ideal models
Liston et al. [50] (2016)	ML/MM ML/MP	Melting Eutectic Peritectic Metatectic Other transitions	DSC curves SLE data Phase diagram Tammann plots	n/a
Moura-Nickel et al. [51] (2016)	EP/Hexadecane ES/Hexadecane EP/ES EP/ES/Hexadecane	Melting	Cloud point data Cloud point diagram	n/a
Robustillo et al. [52] (2016)	EL/DC DC/EP EL/DC/EP	Melting Eutectic Peritectic Other transitions	DSC curves SLE data Phase diagram Tammann plots	Ideal models Flory-Huggins UNIFAC (Dortmund)
Robustillo et al. [53] (2016)	Decane/EL Decane/EP Decane/EL/EP	Melting Eutectic Peritectic Other transitions	DSC curves SLE data Phase diagram Tammann plots	Ideal models Flory-Huggins UNIFAC (Dortmund)
Saeed et al. [54] (2017)	MP/LA	Melting	DSC curves SLE data Thermal conduct. Specific heat Thermal diffusivity Density Cycling stability	Ideal models
Chabane et al. [55] (2018)	EM/Tetradecane EM/Hexadecane EP/Octadecane	Melting Eutectic Peritectic Other transitions	DSC curves SLE data Phase diagram Tammann plots	UNIFAC NRTL Wilson UNIQUAC Ideal models
Maximo et al. [56] (2018)	EP/MP ES/MP EO/MP	Melting Eutectic Peritectic Metatectic Other transitions	Phase diagram Optical images	n/a
Robustillo et al. [57] (2018)	EO/Hexadecane EL/Hexadecane EM/Hexadecane EP/Hexadecane ES/Hexadecane	Melting Eutectic Peritectic Metatectic Other transitions	SLE data Phase diagram Tammann plots	UNIFAC (Dortmund)
Bessa et al. [58] (2019)	EL/Dodecane EM/Dodecane EP/Dodecane ES/Dodecane EO/Dodecane	Melting Eutectic Peritectic Metatectic Other transitions	SLE data Phase diagram Tammann plots	Ideal models UNIFAC (Dortmund) Flory-Huggins
Bessa et al. [59] (2019)	EL/p-Xylene EM/p-Xylene EP/p-Xylene ES/p-Xylene EO/p-Xylene	Melting Eutectic Other transitions	SLE data Phase diagram Tammann plots	Ideal model UNIFAC (Dortmund) Flory-Huggins
Branco et al. [36] (2020)	MS/Hexadecane MS/Octadecane MS/Eicosane MP/Eicosane MP/Hexadecane MP/Octadecane	Melting Eutectic Metatectic Other transitions	Phase diagram Tammann plots XRD spectra	Ideal models

**Table 2:** References grouped by the main purpose of the study.

Purpose of the Study.	References
Study of the behavior of the mixture	[38,39,40]
Data measurement	[30]
Group interaction statistics	[27,28]
Characterization of PCM	[29,48,50,54]
Study of the behavior of biodiesel	[31,32,34,35,36,37,41,42,43,44,45,46,47,49,51,52,53,55,56,57,58,59]

**Table 3:** References reported in this paper, ordered ascendingly according to the eutectic temperature. Numbers marked with an asterisk (*) have been approximately estimated from a graph in the original paper.

Mixture	Eutectic Temp. (K)	Eutectic Enthalpy	References	Comments
MP/MLi	220.00 *	-	[31]	Data of pure MLi
MS/MLi	220.00 *	-	[31]	Data of pure MLi
MO/MLi	220.00 *	-	[31]	Data of pure MLi
ELi/ES	220.68	-	[34]	Data of pure ELi
EP/ELi	220.68	-	[42]	Data of pure ELi
ECa/ELi	220.68	-	[49]	Data of pure ELi
EL/ELi	220.68	-	[49]	Data of pure ELi
EM/ELi	220.68	-	[49]	Data of pure ELi
ECa/ECy	230.35	23 * (kJ/mol)	[49]	Data of pure ECy
ECy/ES	230.35	-	[34]	Data of pure ECy
ECy/EP	230.35	-	[42]	Data of pure ECy
EL/ECy	230.35	27 * (kJ/mol)	[49]	Data of pure ECy
EM/ECy	230.35	20 * (kJ/mol)	[49]	Data of pure ECy
MO/MP	237.15	144 (J/g)	[29]	Data of pure MO
	258.00 *	-	[31]	Data of pure MO
MO/MS	237.15	144 (J/g)	[29]	Data of pure MO
	258.00 *	-	[31]	Data of pure MO
EL/Decane	243.86	27 * (kJ/mol)	[53]	-
EP/Decane	244.63	28 * (kJ/mol)	[53]	Data of pure Decane
ECa/EO	243.89	-	[49]	-
EO/p-Xylene	247.70	35 * (kJ/mol)	[59]	-
EO/Dodecane	250.00	45 * (kJ/mol)	[58]	-
EL/ECa	250.25	28 * (kJ/mol)	[49]	-
EO/EL	250.30	-	[35]	-
EO/MP	251.08	29 * (kJ/mol)	[56]	Data of pure EO
EO/ES	252.35	-	[47]	Data of pure EO
	254.61	-	[34]	Data of pure EO
EO/EP	252.55	-	[35]	Data of pure EO
	254.67	-	[42]	Data of pure EO
EO/EM	252.79	-	[47]	Data of pure EO
EO/Hexadecane	254.00	47 * (kJ/mol)	[57]	Data of pure EO
EM/ECa	252.15	35 * (kJ/mol)	[49]	-
ECa/ES	254.51	-	[34]	Data of pure ECa
ECa/EP	254.51	-	[42]	Data of pure ECa
EL/Dodecane	259.20	26 * (kJ/mol)	[58]	-
EL/p-Xylene	260.10	27 * (kJ/mol)	[59]	-
EM/Dodecane	262.30	34 * (kJ/mol)	[58]	-
EM/Benzene	264.04	-	[30]	-
EP/Dodecane	264.90	43 * (kJ/mol)	[58]	Data of pure dodecane
ES/Dodecane	264.90	43 * (kJ/mol)	[58]	Data of pure dodecane
EL/EM	266.29	35 * (kJ/mol)	[46]	-
	267.00 *	-	[32]	-
	267.90	-	[37]	-
EL/EP	268.69	-	[42]	-
	269.16	-	[35,45,46]	-
	269.80	-	[37]	-
EL/Hexadecane	268.80	23 * (kJ/mol)	[57]	-
EL/DC	269.09	37 * (kJ/mol)	[52]	-
EM/p-Xylene	269.50	25 * (kJ/mol)	[59]	-
	270.13	-	[30]	-
ML/MP	272.00 *	-	[31]	-
	275.01	190.10 (J/g)	[50]	-
EL/ES	272.00 *	-	[32]	-
	272.23	40 * (kJ/mol)	[45]	Data of pure EL
	272.51	-	[34]	Data of pure EL
ML/MM	273.36	174.30 (J/g)	[50]	-
EM/Tetradecane	274.10	126 * (J/g)	[55]	-
EP/p-Xylene	276.10	25 * (kJ/mol)	[59]	-
ES/p-Xylene	280.30	23 * (kJ/mol)	[59]	-
DC/EP	281.67	42 * (kJ/mol)	[52]	-
EM/EP	281.70	20 * (kJ/mol)	[46]	-
	282.60	-	[37]	-
	282.87	-	[42]	-
EM/Hexadecane	281.75	205 * (J/g)	[55]	-
	282.42	42 * (kJ/mol)	[57]	-
EM/ES	282.07	-	[47]	-
	283.37	-	[34]	-
MM/MP	284.65 *	-	[40]	-
	285.00 *	-	[31]	-
	286.52	43 * (kJ/mol)	[41]	-
	287.00 *	-	[32]	-
MM/MS	287.35	45 * (kJ/mol)	[41]	-
EP/Hexadecane	287.58	-	[51]	-
ES/Hexadecane	288.63	-	[51]	-
	288.80	-	[28]	-
	290.38	40 * (kJ/mol)	[57]	-
MP/Hexadecane	289.00	-	[28]	-
MS/Hexadecane	289.00	-	[36]	-
	289.30	-	[28]	-
MN/Hexadecane	289.30	-	[28]	-
EP/MP	293.15	175 (J/g)	[29]	-
	294.10	15 * (kJ/mol)	[56]	-
EP/MS	294.15	178 (J/g)	[29]	-
EP/ES	294.15	209 (J/g)	[29]	-
	294.37	-	[51]	-
	294.97	-	[34]	-
EP/Octadecane	294.45	180 * (J/g)	[55]	-
MP/MS	295.00 *	-	[31]	-
	297.71	41 * (kJ/mol)	[41]	-
	298.15	194 (J/g)	[29]	-
	298.60	-	[39]	-
MP/Eicosane	295.00	-	[36]	-
	295.75	225 * (J/g)	[43]	-
MP/Octadecane	296.00	-	[36]	-
MP/Naphthalene	296.30	-	[44]	-
MP/Biphenyl	296.51	-	[44]	-
ES/MP	297.15	20 * (kJ/mol)	[56]	-
	298.15	171 (J/g)	[29]	-
MS/Octadecane	298.00	-	[36]	-
MP/ME	298.60	-	[39]	-
MP/LA	298.85	205.5 (J/g)	[54]	-
ES/Octacosane	303.90	-	[27]	-
MS/ES	305.15	200 (J/g)	[29]	-
MS/Biphenyl	305.24	-	[44]	-
MS/Naphthalene	306.00	-	[44]	-
MS/Tetracosane	307.03	125 * (J/g)	[43]	-
MS/Octacosane	308.80	-	[27]	-
	309.31	210 * (J/g)	[43]	-

**Table 4:** References described in Section 2.1, ordered by the two compounds of the mixture ^1^.

	ELi	EO	ES	EP	EM	EL	ECa	ECy
**ECy**	-	-	[34]	[42]	[49]	[49]	[49]	
**ECa**	[49]	[49]	[34]	[42]	[49]	[49]		
**EL**	[49]	[35]	[32,34,45]	[32,35,37,42,45,46]	[32,37,46]			
**EM**	[49]	[47]	[34,47]	[37,42,46]				
**EP**	[42]	[35,42]	[29,34,38,45,51]					
**ES**	[34]	[34,47]						
**EO**	-							
**ELi**								

^1^ The meaning of each of the abbreviations of the compounds used in this table can be found in the section “Abbreviations” at the end of the article.

**Table 5:** References described in Section 2.2, ordered by the two compounds of the mixture ^1^.

	ME	MLi	MO	MH	MS	MP	MM	ML
**ML**	-	-	-	-	-	[31,50]	[50]	
**MM**	-	-	-	-	[41]	[31,32,40,41]		
**MP**	[39]	[31]	[29,31]	[39]	[29,31,38,39,41,48]			
**MS**	-	[31]	[29,31]	-				
**MH**	-	-	-					
**MO**	-	[31]						
**MLi**	-							
**ME**								

^1^ The meaning of each of the abbreviations of the compounds used in this table can be found in the section “Abbreviations” at the end of the article.

**Table 6:** References described in Section 2.3, ordered by the two compounds of the mixture ^1^.

	MP	MS
**EP**	[29,56]	[29]
**ES**	[29,56]	[29,38]
**EO**	[56]	-

^1^ The meaning of each of the abbreviations of the compounds used in this table can be found in the section “Abbreviations” at the end of the article.

**Table 7:** References described in Section 2.4, ordered by the two compounds of the mixture ^1^.

	EL	EM	EP	ES	EO	MP	MS	MN
**Decane**	[53]	-	[53]	-	-	-	-	-
**Dodecane**	[58]	[58]	[58]	[58]	[58]	-	-	-
**Tetradecane**	-	[55]	-	-	-	-	-	-
**Hexadecane**	[57]	[55,57]	[51,57]	[28,51,57]	[57]	[28,36]	[28,36]	[28]
**Octadecane**	-	-	[55]	-	-	[36]	[36]	-
**Octacosane**	-	-	-	[27]	-	-	[27,43]	-
**Eicosane**	-	-	-	-	-	[36,43]	[36]	-
**Tetracosane**	-	-	-	-	-	-	[43]	-
**Dodecylcyclohexane**	[52]	-	[52]	-	-	-	-	-
**Biphenyl**	-	-	-	-	-	[44]	[44]	-
**Naphthalene**	-	-	-	-	-	[44]	[44]	-
**Benzene**	-	[30]	-	-	-	-	-	-
**P-Xylene**	[59]	[30,59]	[59]	[59]	[59]	-	-	-
**Lauric acid**	-	-	-	-	-	[54]	-	-

^1^ The meaning of each of the abbreviations of the compounds used in this table can be found in the section “Abbreviations” at the end of the article.

## Data Availability

No new data were created or analyzed in this study.

## Abbreviations

The following abbreviations are used in this manuscript:

CP	Cloud Point
DC	Dodecylcyclohexane
DSC	Differential Scanning Calorimetry
ECa	Ethyl Caprate
ECy	Ethyl Caprylate
EL	Ethyl Laurate
ELi	Ethyl Linoleate
EM	Ethyl Myristate
EO	Ethyl Oleate
EP	Ethyl Palmitate
ES	Ethyl Stearate
FAE	Fatty Acid Esters
FAEE	Fatty Acid Ethyl Esters
FAME	Fatty Acid Methyl Esters
FTIR	Fourier-Transform Infrared
LA	Lauric Acid
LTES	Latent Thermal Energy Storage
ME	Methyl Eicosanoate
MH	Methyl Heptadecanoate
ML	Methyl Laurate
MLi	Methyl Linoleate
MM	Methyl Myristate
MN	Methyl Nonadecanoate
MO	Methyl Oleate
MP	Methyl Palmitate
MS	Methyl Stearate
NRTL	Non-Random Two-Liquid model
PCM	Phase Change Material
SLE	Solid–Liquid Equilibrium
TES	Thermal Energy Storage
UNIFAC	UNIQUAC Functional-group Activity Coefficients model
UNIQUAC	Universal Quasichemical model
